# Development of Novel *N*-isopropylacrylamide (NIPAAm) Based Hydrogels with Varying Content of Chrysin Multiacrylate

**DOI:** 10.3390/gels3040040

**Published:** 2017-10-22

**Authors:** Shuo Tang, Martha Floy, Rohit Bhandari, Thomas Dziubla, J. Zach Hilt

**Affiliations:** 1 Department of Chemical and Materials Engineering, University of Kentucky, Lexington, KY 40506, USA; shuo.tang@uky.edu (S.T.); rohit.bhandari@uky.edu (R.B.); thomas.dziubla@uky.edu (T.D.); 2 Department of Chemical Engineering, Kansas State University, Manhattan, KS 66506, USA; mfloy@ksu.edu; 3 Superfund Research Center, University of Kentucky, Lexington, KY 40506, USA

**Keywords:** hydrogel, *N*-isopropylacrylamide, LCST

## Abstract

A series of novel temperature responsive hydrogels were synthesized by free radical polymerization with varying content of chrysin multiacrylate (ChryMA). The goal was to study the impact of this novel polyphenolic-based multiacrylate on the properties of *N*-isopropylacrylamide (NIPAAm) hydrogels. The temperature responsive behavior of the copolymerized gels was characterized by swelling studies, and their lower critical solution temperature (LCST) was characterized through differential scanning calorimetry (DSC). It was shown that the incorporation of ChryMA decreased the swelling ratios of the hydrogels and shifted their LCSTs to a lower temperature. Gels with different ChryMA content showed different levels of response to temperature change. Higher content gels had a broader phase transition and smaller temperature response, which could be attributed to the increased hydrophobicity being introduced by the ChryMA.

## 1. Introduction

Stimuli responsive hydrogels, which are often referred to as intelligent hydrogels, are a type of hydrogel where swelling behavior changes in response to environmental factors such as pH, salt concentration, ionic strength, and temperature, or a combination therein [1,2,3,4]. Among these, pH and temperature responsive gels have gained the greatest attention, and they have been shown to be easily tunable [5]. *N*-isopropylacrylamide (NIPAAm)-based polymers, as one of the most widely studied temperature responsive polymers, have shown great potentials in various fields. NIPAAm-based polymers exhibit a lower critical solution temperature (LCST) at ~32–33 °C in aqueous solution, which can be easily adjusted to physiological temperature through the modification of the hydrophilic/hydrophobic balance in the polymer with comonomers [6,7,8,9,10]. There are numerous applications of the sharp phase transition, which can be a reversible swelling change for crosslinked systems, especially in the designing of a controlled release system e.g., drug delivery, analytical separation and detection [11,12,13,14,15].

Chrysin (5,7-dihydroxyflavone), a naturally occurring flavonoid presents at high levels in honey and propolis, has shown potential pharmacological effects in inhibiting coronary heart disease, stroke, and cancer [16]. As a flavonoid, chrysin has been widely studied both in vitro and in vivo for its anti-inflammatory, antioxidant, antiviral, and immunomodulatory effects [17]. Multiple researchers have shown that chrysin stimulates or inhibits a wide variety of enzyme systems [17,18,19,20]. In this work, chrysin is not studied as biomedical agent, but it is acrylated and used as a hydrophobic comonomer/crosslinker that is incorporated in an NIPAAm-based temperature responsive hydrogel system.

In the present study, a series of temperature responsive hydrogels consisting of NIPAAm and varying amount of ChryMA were developed. The LCST of NIPAAm gel can be easily altered by adjusting the hydrophilic and hydrophobic balance of the network [7,8]. The use of comonomers to adjust the LCST of NIPAAm gels have been well studied, although such studies have focused mostly on the hydrophilic modification of the polymer network. Some of the well-known hydrophilic crosslinkers/comonomers such as acrylic acid (AA) [21,22,23,24,25,26] and methacrylic acid (MA) [3,27,28,29] have been extensively reported in previous publications. However, few have reported on the hydrophobic crosslinkers/comonomers. Most recently, our group reported two sets of NIPAAm gels using hydrophobic crosslinkers curcumin multiacrylate (CMA) and quercetin multiacrylate (QMA), which have shifted the LCST of NIPAAm gel from 33 °C to 30.4 °C and 28.9 °C, respectively [30]. Other groups, such as Gan et al., investigated the phase transition behavior of hydrophobically modified biodegradable hydrogel using poly(ε-caprolactone) dimethacrylate (PCLDMA) and bisacryloylcysatamine (BACy) as the crosslinkers. The LCST was shifted from 32.6 to 30.68 °C, yet the swelling ratio remained high (~20) [31]. In addition, butyl methacrylate (BMA) [32], di-n-propyl acrylamide (DPAM) [33], polystyrene [34], benzo-12-crown-4-acrylamide (PNB12C4) [35], and others have been reported in recent years on the hydrophobic modification of NIPAAm hydrogel. In this work, we present novel NIPAAm hydrogels with varying content of hydrophobic comonomer/crosslinker ChryMA, which acts as a model compound for the successful synthesis of similar hydrophobic/polyphenolic materials for various fields of application.

## 2. Results and Discussion

### 2.1. Characterization of ChryMA

**Figure 1:**
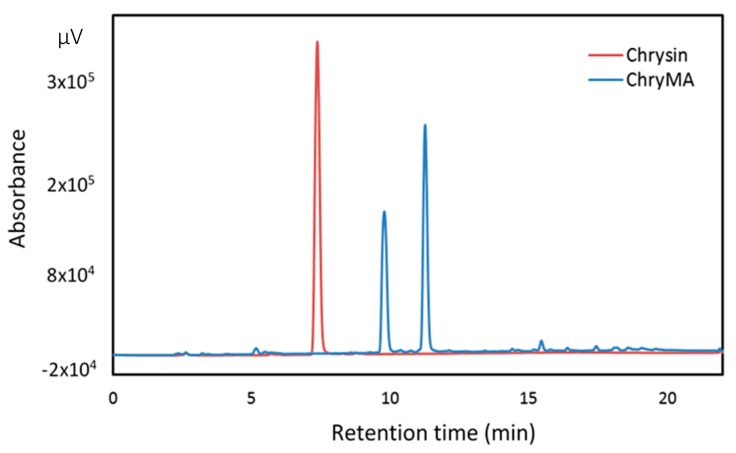
HPLC chromatograms for Chrysin and chrysin multiacrylate (ChryMA).

**Figure 2:**
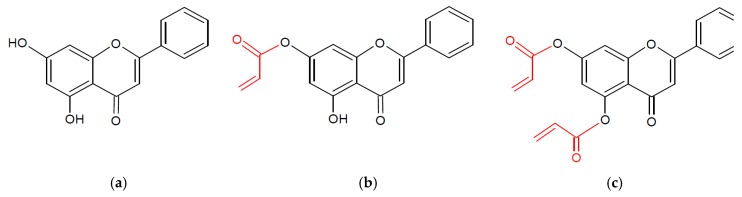
Chemical structure of (**a**) Chrysin; (**b**) Chrysin-monoacrylate; (**c**) Chrysin-diacrylate.

HPLC chromatograms for chrysin and ChryMA are shown in Figure 1. The red and blue lines represent chrysin and ChryMA, respectively. A single and distinctive peak was observed at 7.2 min in the red line, which corresponds to the chrysin with a high purity. Two peaks are shown around 10 and 11 min in blue line, which are the different acrylates of chrysin. On the basis of an increase in the inherent hydrophobicity of the acrylated chrysin, the peak at 10 min was identified as the monoacylate, and the peak at 11 min was identified as the diacrylate. No peak was observed at 7.2 min for the case of ChryMA, indicating that all of the precursor was converted to acrylated product. The composition of mono- and diacrylate was characterized in liquid chromatography time-of-flight (LC-TOF), and these two forms were determined to be present in molar amounts of 36.9% and 63.1%, respectively, which are similar to the observed ratios from HPLC absorbance peak areas. The average molecular weight of ChryMA was calculated to be 365.3 g/mol based on the composition of mono- and diacrylates in the product. The chemical structure of precursor, monoacrylate, and diacrylate are shown in Figure 2.

### 2.2. Synthesis of NIPAAm-co-ChryMA Gels

**Figure 3:**
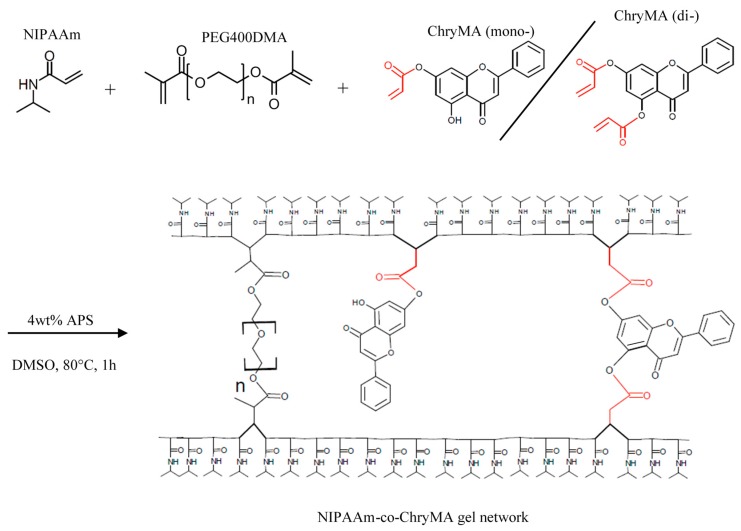
Example polymerization scheme for synthesizing *N*-isopropylacrylamide (NIPAAm)-*co*-ChryMA gels.

NIPAAm-*co*-ChryMA gels were synthesized by free radical polymerization using ammonium persulfate (APS) as a thermal initiator. The crosslinker poly(ethylene glycol) 400 dimethacrylate (PEG400DMA) was kept at 5 mol %, while the ratio of NIPAAm and ChryMA was varied to determine the influence of the hydrophobic crosslinker/comonomer in swelling behaviors and phase transition properties. The control group was synthesized using 5 mol % of PEG400DMA with the rest of NIPAAm, which is referred to as ChryMA 0.0. Three other hydrogel systems were synthesized with 2, 4, or 6 mol % of ChryMA, referred to as ChryMA 2.0, ChryMA 4.0, and ChryMA 6.0, respectively. With these reaction conditions, hydrogels could not be synthesized with more than 6 mol % of ChryMA, which is potentially due to the larger ChryMA molecule sterically hinder gel synthesis at the double bond reactive site. The texture of swollen gel became stiff and rubbery as the amount of ChryMA increased, while low ChryMA content gels were soft and flexible. The polymerization schematic of synthesizing NIPAAm-*co*-ChryMA gel is shown in Figure 3.

### 2.3. Kinetic Swelling Study

**Figure 4:**
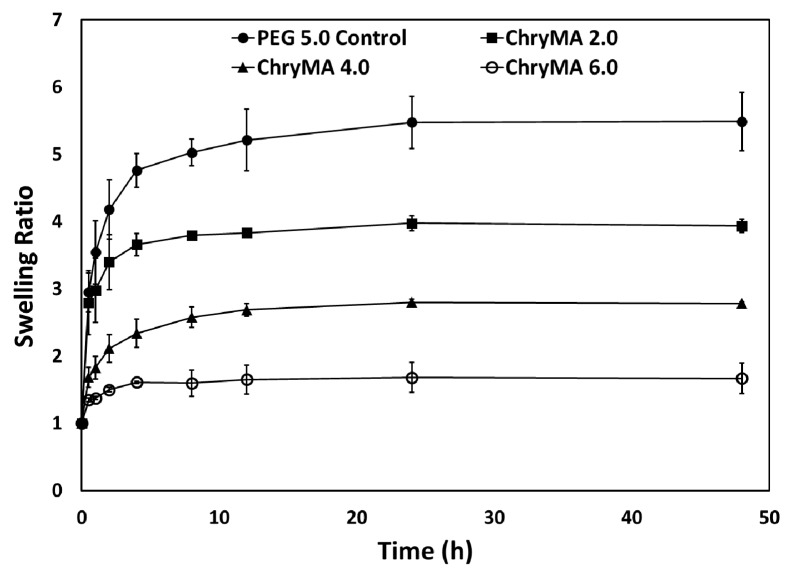
Kinetic swelling behavior of NIPAAm-*co*-ChryMA gels.

The kinetic swelling behavior of NIPAAm-co-ChryMA gels was studied at 25 °C in a water bath for up to 48 h. The mass swelling ratio “*q*” was defined as the mass at the swollen state divided by the mass at the dry state, and the q as a function of time is shown in Figure 4. From the result, it can be noticed that all gels reached equilibrium swelling by 24 h, and the equilibrium swelling ratios decreased with ChryMA content. The addition of ChryMA increases the hydrophobicity of copolymer gels, and thus, the hydrogels have less affinity for water. Furthermore, the increasing ChryMA content in the gel network increases the degree of crosslinking. Both of these factors can be attributed to the lower swelling ratio of the hydrogel with a higher content of ChryMA.

### 2.4. Temperature Dependent Swelling Study

**Figure 5:**
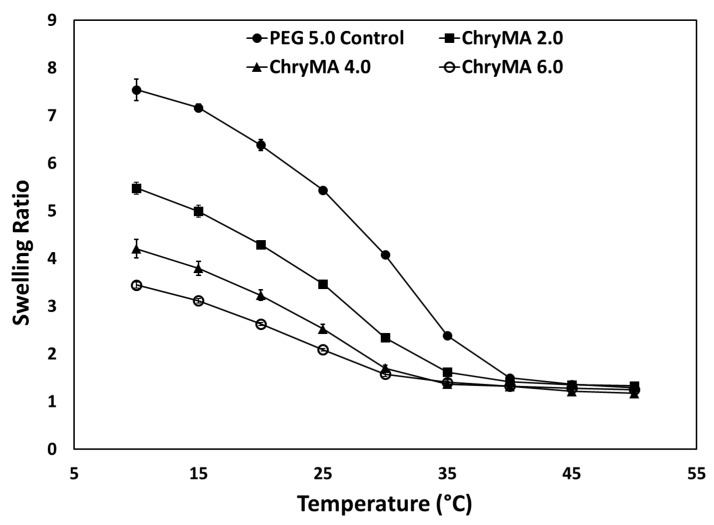
Temperature dependent swelling profile of ChryMA hydrogels.

To study the impact of comonomer content on thermoresponsive swelling behaviors, two temperature dependent swelling studies were conducted. The first study aimed to generate a temperature swelling profile. In this study, copolymer gels were allowed to swell at different temperatures, and their mass swelling ratios were measured after 24 h. As shown in Figure 5, higher temperature caused a lower swelling ratio; and the higher ChryMA content led to a lower transition temperature. Meanwhile, higher ChryMA content gels showed lower swelling and broader phase transition due to more crosslinking in the structure and the addition of hydrophobic content. As temperature increased, all copolymer gels almost completely collapsed by 37.5 °C.

**Figure 6:**
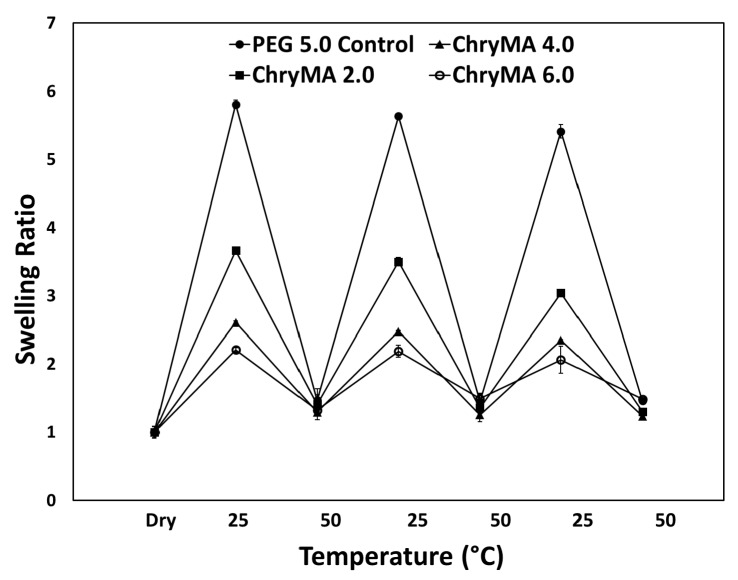
Reversible swelling behavior of NIPAAm-*co*-ChryMA hydrogels.

A second reversible swelling study was also conducted, as shown in Figure 6. The capability of the NIPAAm-*co*-ChryMA hydrogels to swell and deswell repeatedly is important for applications. Reversible temperature changes in this study were designed to span the LCST of the gels going from 10 to 50 °C for ChryMA gels. All gels showed good reversible swelling behavior, retaining swelling within 5%. One possible reason for the slight decrease in the reswelling ratio is the additional crosslink formation through repeated heating. Another reason could be that small fragments of the hydrogel samples were lost during sample handling (especially when they were swollen and fragile), as this could also contribute to the slight decrease in the swelling ratios. Additionally, bubbles formed in ChryMA 6.0 content gels during the heating cycle of the reversible swelling test, and this is likely because the outside thin “skin” barrier is more permeable to water and collapses quickly when undergoing a fast temperature transition. After this outside network collapses, trapped water inside cannot leave the hydrogel and forms a bubble. Zhang et al. reported a similar deswelling and reswelling phenomenon for higher comonomer content gels [36].

### 2.5. LCST Measurements

Various methods are known for determining the LCST of NIPAAm hydrogels, but the most widely used methods are differential scanning calorimetry (DSC) and the equilibrium swelling ratio method. The DSC technique is often more precise since it gives information on the heat released from the cleavage of hydrogen bonds between water and the polymer chain [37,38]. In a typical DSC thermogram, the endothermic peak is referred to as the LCST of the hydrogel, where intramolecular hydrogen bonds between water and NH-break.

Homopolymer NIPAAm is known to have an LCST around 32–33 °C. PEG 5.0 control gel showed an upward shift in LCST to 34.5 °C, which is likely due to the hydrophilicity of PEG400DMA. The incorporation of hydrophilic groups increases the amount of intermolecular hydrogen bonding, so that additional heat is necessary to break the hydrogen bonds, resulting in an increase in the LCST.

**Figure 7:**
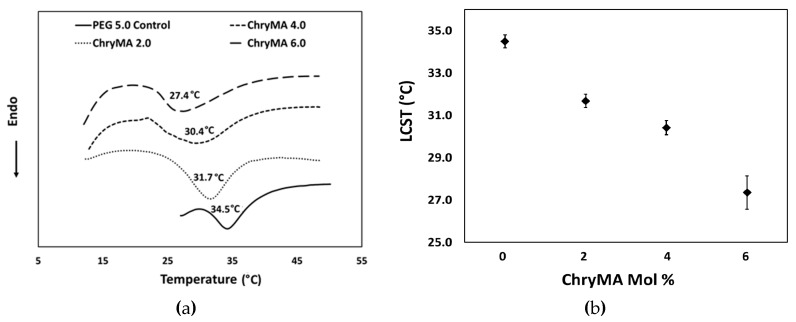
(**a**) Lower critical solution temperature (LCST) measurement of NIPAAm-*co*-ChryMA gels; (**b**) LCST as a function of ChryMA content.

The NIPAAm-*co*-ChryMA gels exhibit an LCST that can be tuned from 34.5 to 27.4 °C by altering the ChryMA composition, as shown in Figure 7. The addition of hydrophobic ChryMA shifted the LCST to lower temperatures due to the fewer hydrogen bonds, meaning that less energy was needed to beak the bonds, thus the transition temperature decreased. In addition, broader peaks were observed for high ChryMA content gels. Important characteristics of synthesized gels are summarized in Table 1.

## 3. Conclusions

Hydrophobically modified NIPAAm-*co*-ChryMA hydrogels were successfully developed through free radical polymerization. Gels with four different compositions were synthesized to study the effect of comonomer content on their swelling and phase transition behaviors. The swelling ratios of copolymeric gels decreased with an increase in ChryMA content, and the addition of ChryMA shifted the LCST to lower temperatures. As temperature increased, swelling decreased for all gels, displaying strong temperature responsive behavior, and this response was shown to be reversible. These novel hydrogels have various potential applications in biomedical, environmental, and other fields.

## 4. Materials and Methods

### 4.1. Materials

*N*-isopropylacrylamide (NIPAAm, 97%), initiator ammonia persulfate (APS, ≥98%) triethyl amine (TEA), acryloyl chloride (AC), and chrysin were purchased from Sigma-Aldrich Corporation (St. Louis, MO, USA). Poly(ethyleneglycol) 400 dimethacrylate (PEG400DMA) was purchased from Polysciences, Inc (Warrington, FL, USA). All solvents were purchased from VWR International (Radnor, PA, USA). Molecular sieves (3 Å) were added to the solvents to remove any moisture present and to maintain their anhydrous state.

### 4.2. Synthesis of ChryMA

Comonomer chrysin multiacrylates (ChryMA) were prepared in accordance with the protocols described previously [39,40]. Briefly, chrysin was dissolved in an excess amount of THF, followed by the addition of AC with the ratio to chrysin of 3:1. TEA was added in the same molar ratio as AC to capture the byproduct hydrogen chloride by forming a chloride salt with the progression of the reaction. AC was added dropwise under continuous stirring in an ice bath to prevent the reaction from overheating. Since Chrysin is light-sensitive, the acrylation process was conducted under dark conditions for 16 h. Then, the obtained mixture was filtered from salts and evaporated under vacuum using a liquid N_2_ trap. Further purification was conducted by multiple washes with 0.1 M K_2_CO_3_ and then 0.1 M HCl to remove any unreacted AC and TEA. The final product was filtrated once more and vacuum dried to obtain powdered ChryMA. The product was kept in a freezer at −20 °C until use.

### 4.3. Characterization of ChryMA Using High Performance Liquid Chromatography (HPLC)

The obtained ChryMA was analyzed through reverse-phase HPLC (Waters Phenomenex C18 column, 5 µm, 250 mm (length) × 4.6 mm (inside diameter, I.D.) on a Shimadzu Prominence LC-20 AB HPLC system) to verify product quality. Samples were dissolved in acetonitrile (ACN) at 100 µg/mL. A gradient from 50/50 ACN/water to 100/0 ACN/water over 24 min at 1 mL/min was used with the column chamber set at 40 °C. The absorbance was measured from 260 nm to 370 nm.

### 4.4. Synthesis of NIPAAm-co-ChryMA Gels 

NIPAAm-*co*-ChryMA hydrogels were synthesized using the free radical polymerization approach. ChryMA and NIPAAm were dissolved in DMSO with the feed ratio of 2/93, 4/91, or 6/89 mol %, and PEG400DMA (crosslinker) was kept at 5 mol %. The initiator, APS, was dissolved in deionized (DI) water to the specified concentration of 0.5 mg/mL and added at 4 wt % combined weight of NIPAAm and ChryMA. To increase solubility, the reaction mixture was preheated at 80 °C before adding the initiator, APS. Instant polymerization occurred, but gels were allowed to continue the reaction for 1 h to ensure high conversion. To remove any unreacted monomers, gels were washed with excess acetone and DI water three times each, for 30 min per wash. Then, gels were cut into small pieces (5 mm in diameter) and freeze-dried overnight until no further mass change occurred. Reaction components for all gels are summarized in Table 1.

### 4.5. Kinetic Swelling Study

Dried gels were swelled in 5 mL of DI water at 25 °C in an isothermal water bath to study equilibrium swelling kinetics. Mass measurements were taken at time points of 0, 0.5, 1, 2, 4, 8, 12, 24, and 48 h. Each sample was removed from the water bath, dabbed dry with Kimwipe to remove excess surface water, and weighed. The mass swelling ratio was defined as the swollen mass divided by the dry mass, as shown in Equation (1), where *M*_swollen_ is the wet weight measured at each time point, and *M*_dry_ is the dry mass after freeze-drying.

(1) $$q\;=\;M_{\text{swollen}}/M_{\text{dry}}$$

### 4.6. Temperature Dependent Swelling Study

To determine the temperature responsiveness of the gels, gels discs were swelled in 5 mL of DI water at different temperatures for 24 h to reach equilibrium swelling. Swelling ratios were measured at temperature increments of 5 °C from 10 to 50 °C. A reversible swelling study was conducted to ensure that the swelling response of the gels was repeatable. Gels were cycled for three times in an isothermal water bath from swollen at 25 °C to collapsed at 50 °C. The mass of the gel was recorded after reaching the swelling equilibrium by 24 h.

### 4.7. LCST Measurements

The LCSTs of the hydrogels were measured using differential scanning calorimetry (DSC Q200, TA instruments Inc., New Castle, DE, USA). Hydrogels were allowed to swell for at least 24 h in DI water for equilibrium. A small piece of gel was gently dabbed dry, and its mass was carefully measured and recorded. The sample was then hermetically sealed in the T-zero pan in order to eliminate the possibility of water evaporation, and placed along with a reference pan on heaters. Samples were heated from 10 to 50 °C at a rate of 2 °C/min under a dry nitrogen atmosphere at a flow rate of 50 mL/min.

## Figures and Tables

**Table 1:** The compositions, equilibrium swelling ratios, and LCSTs of the NIPAAm-*co*-ChryMA gels studied.

Sample	NIPAAm mol %	ChryMA mol %	PEG400DMA mol %	Equilibrium *q* (25 °C)	LCST (°C)
Control	95.0	0.0	5.0	5.5	34.5
ChryMA 2.0	93.0	2.0	5.0	3.8	31.7
ChryMA 4.0	91.0	4.0	5.0	2.6	30.4
ChryMA 6.0	89.0	6.0	5.0	1.7	27.4

## References

[1] Lue S.J., Chen C.-H., Shih C.-M. (2011). Tuning of lower critical solution temperature (LCST) of poly (*N*-isopropylacrylamide-*co*-acrylic acid) hydrogels. J. Macromol. Sci. Part B.

[2] Lee W.-F., Hsu C.-H. (1998). Thermoreversible hydrogels: 3. Synthesis and swelling behavior of the (*N*-isopropylacrylamide-*co*-trimethylacrylamidopropyl ammonium iodide) copolymeric hydrogels. Polymer.

[3] Brazel C.S., Peppas N.A. (1995). Synthesis and Characterization of Thermo-and Chemomechanically Responsive Poly (*N*-isopropylacrylamide-*co*-methacrylic acid) Hydrogels. Macromolecules.

[4] Kim J.H., Lee S.B., Kim S.J., Lee Y.M. (2002). Rapid temperature/pH response of porous alginate-*g*-poly (N-isopropylacrylamide) hydrogels. Polymer.

[5] Haq M.A., Su Y., Wang D. (2017). Mechanical properties of PNIPAM based hydrogels: A review. Mater. Sci. Eng. C.

[6] Hirokawa Y., Tanaka T. Volume phase transition in a non-ionic gel. Proceedings of the AIP Conference.

[7] Ogata T., Nonaka T., Kurihara S. (1995). Permeation of solutes with different molecular size and hydrophobicity through the poly (vinyl alcohol)-graft-*N*-isopropylacrylamide copolymer membrane. J. Membr. Sci..

[8] Hoffman A.S. (1991). Environmentally sensitive polymers and hydrogels. MRS Bull..

[9] Schild H.G. (1992). Poly (*N*-isopropylacrylamide): Experiment, theory and application. Prog. Polym. Sci..

[10] Kuckling D., Wohlrab S. (2002). Synthesis and characterization of biresponsive graft copolymer gels. Polymer.

[11] Safont B., Vitas A., Peñas F. (2009). Biodegradation of phenol in a draft-tube spouted bed bioreactor with biomass attached to hydrogel particles. Water Resources Management V.

[12] Ali W., Gebert B., Hennecke T., Graf K., Ulbricht M., Gutmann J.S. (2015). Design of thermally responsive polymeric hydrogels for brackish water desalination: Effect of architecture on swelling, deswelling, and salt rejection. ACS Appl. Mater. Interfaces.

[13] Ashraf S., Park H.-K., Park H., Lee S.-H. (2016). Snapshot of phase transition in thermoresponsive hydrogel PNIPAM: Role in drug delivery and tissue engineering. Macromol. Res..

[14] Klouda L. (2015). Thermoresponsive hydrogels in biomedical applications: A seven-year update. Eur. J. Pharm. Biopharm..

[15] Díez-Peña E., Frutos P., Frutos G., Quijada-Garrido I., Barrales-Rienda J.M. (2004). The influence of the copolymer composition on the diltiazem hydrochloride release from a series of pH-sensitive poly [(N-isopropylacrylamide)-co-(methacrylic acid)] hydrogels. AAPS PharmSciTech.

[16] Walle U.K., Galijatovic A., Walle T. (1999). Transport of the flavonoid chrysin and its conjugated metabolites by the human intestinal cell line Caco-2. Biochem. Pharmacol..

[17] Comalada M., Ballester I., Bailón E., Sierra S., Xaus J., Gálvez J., de Medina F.S., Zarzuelo A. (2006). Inhibition of pro-inflammatory markers in primary bone marrow-derived mouse macrophages by naturally occurring flavonoids: Analysis of the structure–activity relationship. Biochem. Pharmacol..

[18] Wolfman C., Viola H., Paladini A., Dajas F., Medina J.H. (1994). Possible anxiolytic effects of chrysin, a central benzodiazepine receptor ligand isolated from Passiflora coerulea. Pharmacol. Biochem. Behav..

[19] Zeng Y.-B., Yang N., Liu W.S., Tang N. (2003). Synthesis, characterization and DNA-binding properties of La (III) complex of chrysin. J. Inorg. Biochem..

[20] Nielsen S., Breinholt V., Justesen U., Cornett C., Dragsted L.O. (1998). In vitro biotransformation of flavonoids by rat liver microsomes. Xenobiotica.

[21] Xue W., Champ S., Huglin M.B. (2001). Network and swelling parameters of chemically crosslinked thermoreversible hydrogels. Polymer.

[22] Velada J.L., Liu Y., Huglin M.B. (1998). Effect of pH on the swelling behaviour of hydrogels based on *N*-isopropylacrylamide with acidic comonomers. Macromol. Chem. Phys..

[23] Yoo M., Sung Y.K., Lee Y.M., Cho C.S. (2000). Effect of polyelectrolyte on the lower critical solution temperature of poly (*N*-isopropyl acrylamide) in the poly (NIPAAm-co-acrylic acid) hydrogel. Polymer.

[24] Kim S., Healy K.E. (2003). Synthesis and characterization of injectable poly (*N*-isopropylacrylamide-*co*-acrylic acid) hydrogels with proteolytically degradable cross-links. Biomacromolecules.

[25] Chen G., Hoffman A.S. (1995). Temperature-induced phase transition behaviors of random vs. graft copolymers of *N*-isopropylacrylamide and acrylic acid. Macromol. Rapid Commun..

[26] Jones M. (1999). Effect of pH on the lower critical solution temperatures of random copolymers of *N*-isopropylacrylamide and acrylic acid. Eur. Polym. J..

[27] Zhang J., Peppas N.A. (2000). Synthesis and characterization of pH-and temperature-sensitive poly (methacrylic acid)/poly (*N*-isopropylacrylamide) interpenetrating polymeric networks. Macromolecules.

[28] Qiu Y., Park K. (2001). Environment-sensitive hydrogels for drug delivery. Adv. Drug Deliv. Rev..

[29] Brazel C.S., Peppas N.A. (1996). Pulsatile local delivery of thrombolytic and antithrombotic agents using poly (*N*-isopropylacrylamide-co-methacrylic acid) hydrogels. J. Control. Release.

[30] Tang S., Bhandari R., Delaney S.P., Munson E.J., Dziubla T.D., Hilt J.Z. (2017). Synthesis and characterization of thermally responsive *N*-isopropylacrylamide hydrogels copolymerized with novel hydrophobic polyphenolic crosslinkers. Mater. Today Commun..

[31] Gan J., Guan X.X., Zheng J., Guo H., Wu K., Liang L., Lu M. (2016). Biodegradable, thermoresponsive PNIPAM-based hydrogel scaffolds for the sustained release of levofloxacin. RSC Adv..

[32] Feil H., Bae Y.H., Feijen J., Kim S.W. (1993). Effect of comonomer hydrophilicity and ionization on the lower critical solution temperature of *N*-isopropylacrylamide copolymers. Macromolecules.

[33] Xue W., Hamley I.W. (2002). Thermoreversible swelling behaviour of hydrogels based on *N*-isopropylacrylamide with a hydrophobic comonomer. Polymer.

[34] Singh R., Deshmukh S.A., Kamath G., Sankaranarayanan S.K.R.S., Balasubramanian G. (2017). Controlling the aqueous solubility of PNIPAM with hydrophobic molecular units. Comput. Mater. Sci..

[35] Wei Y.Y., Liu Z., Ju X., Shi K., Xie R., Wang W., Cheng Z., Chu L. (2016). Gamma-Cyclodextrin-Recognition-Responsive Characteristics of Poly (N-isopropylacrylamide)-Based Hydrogels with Benzo-12-crown-4 Units as Signal Receptors. Macromol. Chem. Phys..

[36] Zhang X.-Z., Sun G.M., Wu D.Q., Chu C.C. (2004). Synthesis and characterization of partially biodegradable and thermosensitive hydrogel. J. Mater. Sci. Mater. Med..

[37] Constantin M., Cristea M., Ascenzi P., Fundueanu G. (2011). Lower critical solution temperature versus volume phase transition temperature in thermoresponsive drug delivery systems. Express Polym. Lett..

[38] Boutris C., Chatzi E., Kiparissides C. (1997). Characterization of the LCST behaviour of aqueous poly (*N*-isopropylacrylamide) solutions by thermal and cloud point techniques. Polymer.

[39] Gupta P., Jordan C.T., Mitov M.I., Butterfield D.A., Hilt J.Z., Dziubla T.D. (2016). Controlled curcumin release via conjugation into PBAE nanogels enhances mitochondrial protection against oxidative stress. Int. J. Pharm..

[40] Gupta P., Authimoolam S.P., Hilt J.Z., Dziubla T.D. (2015). Quercetin conjugated poly (β-amino esters) nanogels for the treatment of cellular oxidative stress. Acta Biomater..

